# Golgi fragmentation precedes neuromuscular denervation and is associated with endosome abnormalities in SOD1-ALS mouse motor neurons

**DOI:** 10.1186/2051-5960-2-38

**Published:** 2014-04-07

**Authors:** Vera van Dis, Marijn Kuijpers, Elize D Haasdijk, Eva Teuling, Scott A Oakes, Casper C Hoogenraad, Dick Jaarsma

**Affiliations:** 1 Department of Neuroscience, Erasmus Medical Center, P.O.Box 2040, 3000 CA Rotterdam, The Netherlands; 2 Cell Biology, Faculty of Science, Utrecht University, Utrecht, The Netherlands; 3 Department of Pathology, University of California, San Francisco, USA

**Keywords:** Amyotrophic Lateral Sclerosis (ALS), Protein aggregation, Endosome trafficking, Dynein, Transgenic mouse model, SOD1

## Abstract

**Background:**

Fragmentation of stacked cisterns of the Golgi apparatus into dispersed smaller elements is a feature associated with degeneration of neurons in amyotrophic lateral sclerosis (ALS) and some other neurodegenerative disorders. However, the role of Golgi fragmentation in motor neuron degeneration is not well understood.

**Results:**

Here we use a SOD1-ALS mouse model (low-copy Gurney G93A-SOD1 mouse) to show that motor neurons with Golgi fragmentation are retrogradely labeled by intramuscularly injected CTB (beta subunit of cholera toxin), indicating that Golgi fragmentation precedes neuromuscular denervation and axon retraction. We further show that Golgi fragmentation may occur in the absence of and precede two other pathological markers, i.e. somatodendritic SOD1 inclusions, and the induction of ATF3 expression. In addition, we show that Golgi fragmentation is associated with an altered dendritic organization of the Golgi apparatus, does not depend on intact apoptotic machinery, and is facilitated in transgenic mice with impaired retrograde dynein-dependent transport (BICD2-N mice). A connection to altered dynein-dependent transport also is suggested by reduced expression of endosomal markers in neurons with Golgi fragmentation, which also occurs in neurons with impaired dynein function.

**Conclusions:**

Together the data indicate that Golgi fragmentation is a very early event in the pathological cascade in ALS that is associated with altered organization of intracellular trafficking.

## Introduction

Death and disappearance of neurons is a constant hallmark of neurodegenerative disorders, such as Alzheimer's disease and other dementia's, Parkinson's disease, and amyotrophic lateral sclerosis (ALS). A large body of evidence indicates that protein aggregation and proteotoxicity are central early pathogenic steps in these disorders, but how these events ultimately result in neuronal malfunction and degeneration is yet poorly established for most disorders [1,2]. Various mechanisms involving improper function of specific organelles, in particular mitochondria and the endoplasmic reticulum (ER), or disrupted function of cellular processes such as protein quality control, intracellular trafficking, cell death signaling, synaptic signaling or calcium homeostasis have been proposed to contribute to neuronal degeneration [2-5]. Neuropathological studies have also revealed abnormalities in the Golgi apparatus in a variety of neurodegenerative diseases [6-8]. The Golgi apparatus is a highly dynamic organelle involved in processing and sorting of lipids and proteins. Its morphology depends on a large variety of protein components and cellular processes [9-13]. Accordingly, Golgi morphology can alter under a variety of physiological conditions such as cell division, growth, and altered metabolic demands [9], as well as pathological conditions, including impaired ER function, disruption of intracellular transport, altered lipid metabolism, excessive excitation, DNA damage, and activation of cell death pathways [6,14-19].

Golgi fragmentation is a frequent feature in motor neurons of ALS patients, where it may occur in 10-50% of motor neurons in sporadic and SOD1-ALS patients [6,20,21] and up to 70% of the motor neurons in some patients with familial ALS and ALS-like disorders [22,23]. The occurrence of Golgi fragmentation correlates with nuclear-to-cytoplasmic redistribution of TDP-43 and the presence of TDP-43 positive inclusions in sporadic ALS motor neurons [24]. Golgi fragmentation also correlates with the presence of inclusions in other ALS forms including optineurin-ALS and SOD1-ALS [21-23], but its role in motor neuron degeneration is not understood.

Golgi fragmentation also occurs in transgenic ALS rodent models [25-27]. It has been identified as an early feature in motor neurons in transgenic mice that express human SOD1 with an ALS-linked mutation and develop an ALS like motor neuron disorder [27-29]. To further understand the pathological significance of Golgi fragmentation in ALS, in the present study we have precisely characterized the relationship between Golgi fragmentation and other early pathological events in the SOD1-ALS mouse model. Our data show that Golgi fragmentation precedes the appearance of somatodendritic SOD1 inclusions, activation of the injury transcription factor ATF3, neuromuscular denervation and axon retraction. We further show that Golgi fragmentation also occurs in the absence of a functional mitochondrial apoptotic pathway, is greatly facilitated in neurons with impaired dynein function and is associated with endosomal abnormalities. These data suggest that Golgi fragmentation reflects altered organization of intracellular trafficking, perhaps in response to specific aggregated SOD1 complexes.

## Materials and methods

### Transgenic mice

All animal experiments were approved by the Erasmus University animal care committee, and performed in accordance with the guidelines the "Principles of laboratory animal care" (NIH publication No. 86–23), and the European Community Council Directive (86/609/EEC). Transgenic mice expressing a human genomic SOD1 construct with the G93A mutation were originally derived from the Gurney G1 mice, but because of a reduction in the transgene copy number (8 instead of ~20 transgene copy numbers per haploid genome) show a delayed disease onset and are termed G1del mice [30-32]. G1del mice are maintained under standard housing conditions in a FVB/N background by mating hemizygote males with non-transgenic females, non-transgenic offspring serving as controls [31].

Neuron-specific mice carrying the cDNA of G93A-mutant hSOD1 cloned into the Thy1.2 expression cassette were generated as described [31]. In this study we used tissue from T3hSOD1 double transgenic mice generated by crossing Thy1hSOD1-G93A mice derived from founder T3 with line N1029 wild-type hSOD1 overexpressing mice [31].

BICD2-N (line BN1) mice are transgenic for a construct expressing the N-terminus of Bicaudal D2 coupled to GFP (GFP-BICD2-N) and cloned into the Thy1.2 expression vector [17]. BN1/G1del mice were obtained by intercrossing hemizygous G1del and BN1 mice as described [17]. In this study we analyzed Golgi fragmentation in spinal cord sections from G1del, G1del/BN1 and BN1 killed at 28 weeks of age with 4–6 mice per genotype. Both G1 del and G1del/N1 mice showed mild-to-moderate muscle weakness of hind limb function.

For one experiment we used paraffin embedded spinal cord tissue from 90 days old high copy (fast) G1 mice (carrying ~20 hSOD1-G93A transgene copy numbers, see above) crossed with mice deficient for the mitochondrial apoptotic pathway in the central nervous system, termed DKO mice. DKO mice were Bak-null in the whole body (*Bak*^*-/-*^) and Bax-null in the central nervous system (*Bax*^*f/f*^*/Nestin-Cr*e; see [33] for details). We analyzed archival sections from 3 mice per genotype (non-tg, DKO, G1del, G1del/DKO).

### Antibodies

Primary antibodies (supplier; dilutions) used in this study are:

Rabbit anti-ATF3 (Santa Cruz; IHC and IF 1:1000), goat-anti-choline acetyltransferase (ChAT, Millipore, 1:500), goat anti-Cholera toxin B subunit (CTB; List Biological laboratories, 1:5000), rabbit-anti-CGRP (Calbiochem, IF, 1:10.000), human-anti EEA1 (gift from Dr. M.J. Fritzler, IF: 1:500 [34]), mouse-anti GM130 (BD Biosciences, 1:200), rabbit-anti GM130 (antibody M07, gift from Dr. M. Lowe [35], 1:1000), mouse anti GRASP65 (gift from Dr. M. Lowe [35], 1:200), rabbit-anti-GRASP-1 (Millipore, 1:500), rat anti-Mac-2 (Cedarlane, 1:2000), mouse-anti p115 (BD Biosciences, 1:500), sheep anti-hSOD1 (Calbiochem; IF, 1:5000), rabbit-anti unfolded SOD1 (USOD, gift from Dr. A. Chakrabartty [36], 1:1000), rabbit anti-SOD1 exposed dimer interface (SEDI, gift from Dr. J. Robertson [37], 1:1000), mouse anti-ubiquitin (clone FK2; Affinity BioReagents, Golden, CO; 1:2000, rabbit anti-ubiquitin (DAKO, 1:1000).

Secondary antibodies used for IF or ICC were Alexa-488, -568 or -633- conjugated goat-anti mouse or goat-anti rabbit antibodies from Molecular Probes; or FITC, Cy3 or Cy5 donkey-anti-rabbit, donkey-mouse or donkey-goat antibodies from Jackson Laboratories; all antibodies were used at 1:400. Biotinylated goat-anti-rabbit or anti-mouse IgG (Vector) were used at 1:400.

### Immunocytochemical and histochemical analyses

For immunocytochemistry and immunofluorescence, mice were anaesthetized with pentobarbital and perfused transcardially with 4% paraformaldehyde with or without glutaraldehyde (0.05%). The lumbar and cervical spinal cord were carefully dissected out and postfixed overnight in 4% paraformaldehyde. Routinely, spinal cord tissue was embedded in gelatin blocks [38], sectioned at 40 μm with a freezing microtome, and sections were processed, free-floating, using a standard avidin–biotin–immunoperoxidase complex method (ABC, Vector Laboratories, USA) with diaminobenzidine (DAB, 0.05%) as the chromogen, or single-, double- and triple-labelling immunofluorescence. Immunoperoxidase-stained sections were analysed and photographed using a Leica DM-RB microscope and a Leica DC300 digital camera. Sections stained for immunofluorescence were analyzed with a Leica DM-RB epifluorescence microscope and Zeiss LSM 510 and LSM700 confocal laser scanning microscopes.

### Cholera toxin B-fragment (CTB) retrograde tracing

**Figure 1:**
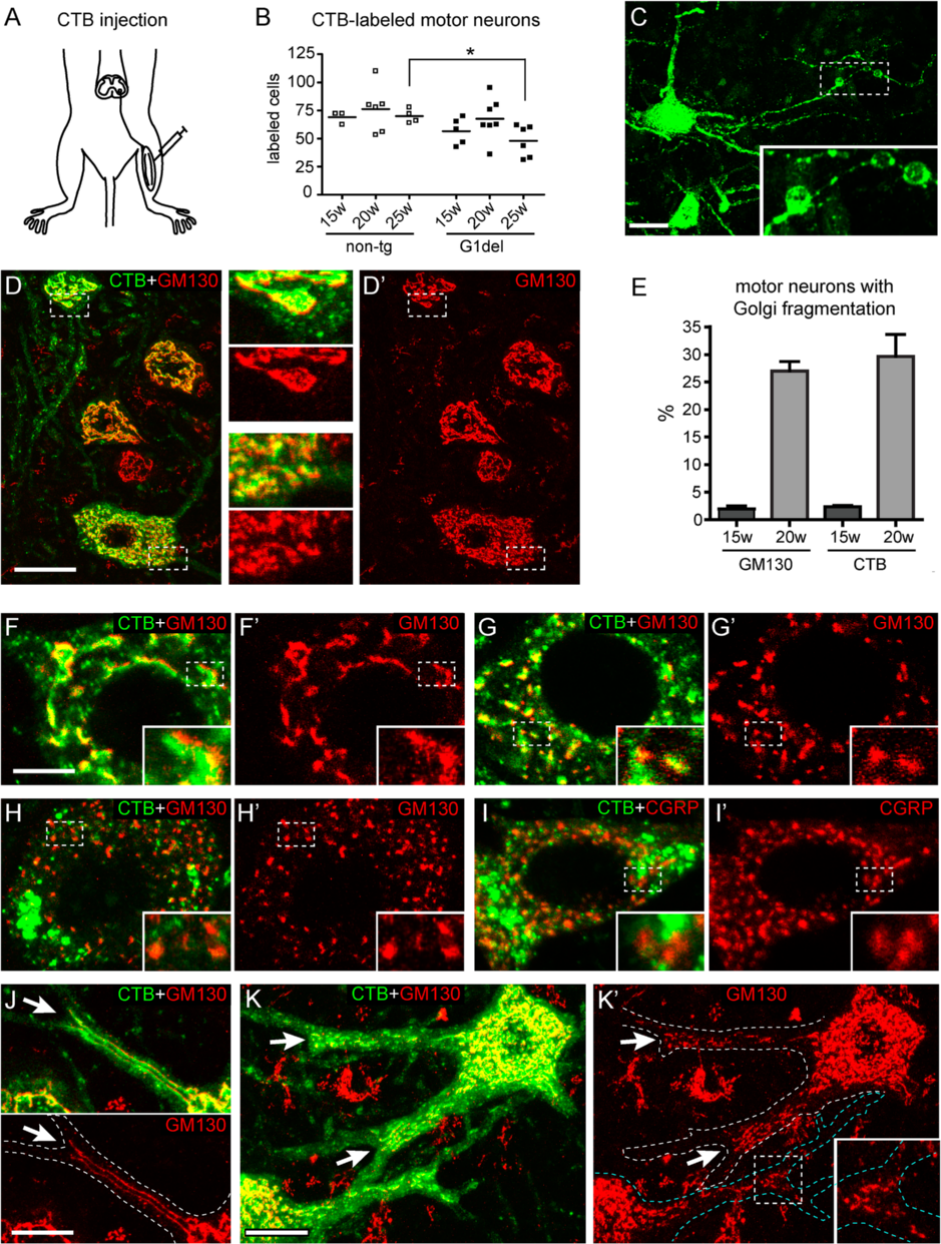
**Retrograde labeling of motor neurons with Golgi fragmentation following intramuscular injections of CTB. A-C)** CTB (0.5% in 2.5 μl saline) is injected in the gastrocnemic muscle to retrogradely label motor neurons **(A)**. **B)** Dot plot showing the number of CTB-positive motor neurons counted in each tenth section. Note reduced number of CTB-positive cells in 25 week old G1del mice compared to age matched non-transgenic mice (P < 0.05, unpaired Student's *t*-test). **C)** Maximal projection of confocal stack (optical section 22 μm) of CTB-labeled motor neuron illustrating small vacuoles in the distal dendrite (insert). **D)** Double-labeling confocal image of CTB and GM130 showing cross sections of 4 retrogradely labeled motor neurons. A large proportion of CTB localizes to the Golgi apparatus. The motor neuron below has fragmented Golgi. **E)** Bar graph showing the proportion of motor neurons with Golgi fragmentation in all large ventral horn neurons (GM130) and the CTB labeled population. F-I) Representative images of a CTB-labeled motor neuron with normal Golgi apparatus **(F)**, fragmented Golgi **(G)**, and fragmented Golgi with no or little CTB **(H, I)**. **J)** Representative example of a proximal motor neuron dendrite with GM130-positve ribbons that stop at the branching point (arrow). **K)** Two motor neurons with Golgi fragmentation showing fragmented Golgi in the proximal dendrites up to the first branching point (arrows and insert). Bars: 20 μm **(C, D)**, 10 μm **(K)**, 5 μm **(F, J)**.

Mice were anaesthetized, immobilized, and Cholera toxin B-fragment (CTB) (0.5% in 2.5 μl saline) was injected in the gastrocnemic muscle of 15, 20 and 25 weeks old G1del mice and non-transgenic littermates (Figure 1A). 4 days following injections mice were perfused with 4% paraformaldehyde with or without glutaraldehyde (0.05%), and their spinal cords were embedded in gelatin blocks, sectioned at 40 μm, and further processed for immunohistological procedures. To obtain an estimate of the number of retrogradely labeled motor neurons, one in 10 sections was immunoperoxidase-DAB stained for CTB and used for counting CTB-positive cells with the aid of an Olympus microscope fitted with a LucividTM miniature monitor and NeurolucidaTM software(MicroBrightField, Colchester, VT, USA). Only CTB-positive cells whose nucleus was partially or entirely present in the section, but did not contact the surface of the sections, were counted.

### Quantitative analysis of immunofluorescence images

The proportion of CTB and GM130-positive motor neurons was determined in sections from G1del mice of 15 (n = 3) and 20 weeks (n = 3), 4 sections/mouse using a Leica DM-RB epifluorescence microscope with a 63x objective. First, all large neurons (diameter >20 μm) in the ventral horn both ipsi- and contralateral of the injection were scored for Golgi fragmentation, and subsequently using the same sections CTB-positive cells were scored for Golgi fragmentation.

Dendritic GM130 labelling was examined with a Zeiss LSM 510 laser scanning microscope using sections from 20 weeks old G1del mice. CTB-labeled motor neurons were selected for analysis on the basis of the presence of at least 3 dendrites within the plane of section, and with at least 2 of these dendrites having the first branching point in the section. Confocal stacks of CTB and GM130 stacks were collected, and cells were analyzed for the occurrence of Golgi fragmentation and the presence of dendritic GM130 labeling. A total of 24 and 14 motor neurons with normal and fragmented Golgi apparatus, respectively, were analyzed (Additional file 1: Table S1).

For analysis of fluorescence intensities, images were collected using a Zeiss LSM 510 confocal laser scanning microscope with 63x objective. Fluorescence intensities were determined using Metamorph image analysis software.

Statistical analyzes were performed with MS Excel or Graphpad Prism software using Student’s *t*-test and one-way ANOVA.

### Primary neuron cultures and transfection

Primary rat hippocampal neurons were plated at a density of 75.000 on 18 mm glass coverslips and transfected at DIV13 with GFP, GFP-p150-cc, GFP-BICD2-N or GFP-p50 using Lipofectamine-2000 (Qiagen) as described [17,39]. After 2 days of transfection, neurons were fixed and stained with human anti-EEA1 antibodies. Representative cells were imaged using a Zeiss LSM 510 confocal laser scanning microscope with a x63 oil immersion objective. The number of particles were analyzed using Metamorph software.

## Results

### Retrograde labeling of motor neurons with Golgi fragmentation after intramuscular injection of cholera toxin B (CTB)

The present study has been mainly performed with G1del mice (also termed G1slow) that carry 8 copies of a human G93A-mutant SOD1 and develop weakness in one or more limbs from age 24–34 weeks of age and die of fatal paralysis between 30–41 weeks of age [29,32]. The onset of Golgi fragmentation in G1del mice is at about 14–15 weeks of age [29]. In view of evidence that degeneration of the distal axon is an early event in SOD1-ALS mice preceding the loss of motor neurons [40-42], we first examined whether motor neurons with Golgi fragmentation have an intact axon. For this purpose, we performed retrograde tracing of Choleratoxin B (CTB) injected in the gastrocnemic muscle (Figure 1A), and double stained lumbar spinal cord sections for CTB and the cis-Golgi matrix protein GM130 [43]. CTB is taken up via endocytosis after binding to the ganglioside GM1 in motor nerve endings and then retrogradely transported to the cell bodies, where it accumulates in the trans-Golgi, lysosomes, and the cytoplasm [44,45]. The number of CTB labeled motor neurons was the same as in non-transgenic littermates in G1del mice at 15 and 20 weeks, and was reduced in G1del mice at 25 weeks (Figure 1B), consistent with the onset of motor neuron loss at 25 weeks [29]. CTB labeling was present throughout the soma and dendrites. Typically, motor neurons of G1del mice showed small vacuolar expansions in the distal portion of the dendrites (Figure 1C), reflecting vacuolated mitochondria [46]. A proportion of CTB-positive cells showed Golgi fragmentation (Figure 1D-K). Importantly, the proportion of retrogradely CTB-labeled motor neurons with Golgi fragmentation is the same as the proportion of Golgi fragmented motor neurons in the total population of motor neurons (Figure 1E). This indicates that Golgi fragmentation does not interfere with retrograde labeling.

In CTB positive motor neurons with a normal Golgi apparatus, a proportion of CTB codistributed with GM130, consistent with retrograde trafficking of CTB to the Golgi apparatus (Figure 1D). Accordingly, also in the majority of motor neurons with Golgi fragmentation, CTB codistributed with GM130 (Figure 1F, G). However, in a subset of CTB-positive motor neurons with Golgi fragmentation, CTB poorly codistributed with GM130 (Figure 1H). In these cells, CTB was distributed in large clusters into the cell body, in lysosomes, and diffusely throughout the somato-dendritic compartment (Figure 1H).

We also performed double-labelling of CTB with αCGRP (calcitonin-gene related peptide), a peptide that is present in the trans-Golgi and secretory granules of most large motor neurons [47]. Consistent with the results of GM130-CTB double labelling, the majority of CTB-positive motor neurons with Golgi fragmentation showed codistribution of CTB and CGRP in the Golgi apparatus, while in a small subset of CTB-positive motor neurons with fragmented Golgi apparatus, CTB poorly colocalized with CGRP (Figure 1I). Together, these data indicate that although motor neurons with a fragmented Golgi can take up, retrogradely transport and redistribute the tracer throughout the somatodendritic compartment, a subset of neurons with fragmented Golgi may have compromised retrograde transport to the Golgi apparatus. The data also indicate that motor neurons with Golgi fragmentation may represent different stages of degeneration varying from near-normal cells to cells in a more progressed degenerative state (see below).

### A larger proportion of dendrites with Golgi apparatus in motor neurons with Golgi fragmentation

The presence of CTB enabled to easily distinguish the proximal dendrites extending from the motor neurons. A subset of motor neurons in both non-transgenic and G1del mice showed one or more GM130-positive ribbons that entered the proximal dendrite and coursed up to the first branching point (Figure 1J). Also motor neurons with Golgi fragmentation showed Golgi apparatus entering the proximal dendrites. In these cells the dendritic Golgi always was fragmented into smaller elements (Figure 1K). Random sampling of motor neurons with 3 or more proximal dendrites extending from the cell body in the plane of section, indicated that motor neurons with Golgi fragmentation more often showed dendrites with Golgi apparatus (10 of 24 versus 12 of 14 analyzed motor neurons with normal or fragmented Golgi apparatus, respectively; see Additional file 1: Table S1). The mean number of dendrites per sampled cell was the same for cells with or without Golgi fragmentation (3.5 ± 0.14 versus 3.7 ± 0.24 for normal or fragmented Golgi apparatus, respectively; non-significant, unpaired student's *t*-test; see Additional file 1: Figure S1). Overall, dendritic Golgi apparatus occurred 3 times more often in motor neurons with fragmented Golgi (Additional file 1: Table S1 and Figure S1). These data suggest that motor neurons with Golgi fragmentation show an increased probability of extending the Golgi apparatus in one or more proximal dendrites.

### Golgi fragmentation precedes the appearance of ubiquitin-positive inclusions in the cell body of motor neurons

**Figure 2:**
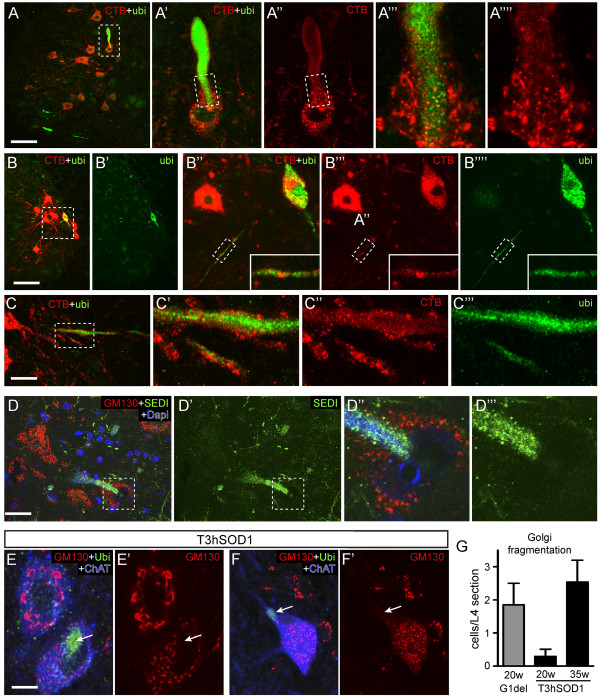
**CTB tracing and Golgi fragmentation in motor neurons with ubiquitinated SOD1-inclusions. A-C)** Double-labeling confocal image of CTB and ubiquitinated epitopes showing a CTB-positive motor neuron with a large inclusion in the proximal dendrite **(A' and A'')**, a CTB-positive motor neuron with intense ubiquitin labeling throughout most of the somato-dendritic compartment **(B' and B'''')**, and ubiquitin-labeling in distal CTB-positive dendrites **(C-C''')**. **D)** Double-labeling confocal image of GM130 and misfolded SOD1 (SEDI epitope) showing a motor neuron with fragmented Golgi and a large SEDI-immunoreactive inclusion. **E-F)** Confocal image showing motor neurons labeled for ChAT with fragmented Golgi apparatus and poly-ubiquitinated epitope (arrow) in L4 lumbar spinal cord section of 20 **(E)** and 35 **(F)** weeks old T3hSOD1 mouse. **G)** Bar graph showing the number of Golgi fragmented motor neurons in L4 spinal cord sections of G1del versus T3hSOD1 mice. Values represent Means ± SE from 3 mice per group. Note that no motor neurons with fragmented Golgi occur in non-transgenic, T3 and hSOD1 mice at 35 weeks of age. Bars: 50 μm **(A-C)**, 10 μm **(D-F)**.

We have previously shown that the onset of Golgi fragmentation in G1del mice grossly coincides with the onset of other pathological signs, i.e. the appearance of somatodendritic ubiquitinated SOD1 inclusions, the induction of stress transcription factor ATF3 expression, and signs of microglia activation at about 14–15 weeks of age [29,31,38]. For comparison, also in the high-copy fast G1 mouse the onset of Golgi fragmentation at post-natal day 30 [27] coincides with the onset of ATF3 expression, signs of ER stress and microglia activation [48]. In G1del mice, ubiquitinated SOD1 inclusions initially and most frequently occur in the dendrites of motor neurons, while inclusions in the cell body are relatively infrequent even in symptomatic animals (>24 weeks) [29,31,38]. In these cells, ubiquitin labelling is associated with compact inclusions that generally extend into a dendrite, or is diffusely distributed throughout the cell body [29]. While, ‘ubiquitinated’ motor neurons are relatively rare and represent only a minority of motor neurons with fragmented Golgi, inversely ubiquitinated motor neurons in all occasions show Golgi fragmentation [29]. In addition, the ubiquitinated motor neurons frequently show a flattened and eccentric nucleus, suggestive of a progressed stage of degeneration [29]. To determine whether the ubiquitinated motor neurons are still capable of retrogradely transporting CTB, we double-labeled spinal cord sections from CTB-injected mice for CTB and ubiquitin. Ubiquitinated motor neurons that were also CTB-positive were identified in sections from 20 and 25 weeks old G1del mice (Figure 2A, B; 13 double-labeled cells in 24 lumbar L4 sections: 4 sections/mouse with 3 mice/age group), but not in sections from 15 weeks old G1 del mice (16 sections from 4 mice; 4 sections/mouse). Ubiquitinated aggregates also occurred in CTB-positive dendrites, sporadically in 15 weeks old mice, and more frequently in 20 and 25 weeks old G1del mice (Figure 2C). These date indicate that even motor neurons with severe ubiquitin pathology may have a relatively intact axon in G1del mice, and that the presence of ubiquitin pathology in more distal dendrites still allows trafficking of CTB to this dendrite.

To analyze the relationship between Golgi fragmentation and the accumulation of misfolded SOD1 species, we performed double labeling of GM130 with antibodies against SOD1 dimer interface (SEDI, [37]) and unfolded beta barrel domain of SOD1 (USOD, [36]). The USOD antibody mainly stained vacuolated mitochondria in the axon and distal dendrites, and, in addition, produced diffuse staining in the cell bodies of motor neurons and lightly stained ubiquitinated inclusions (not shown). The SEDI antibody, apart from light staining of vacuolated mitochondria and some background staining of astrocytic processes, selectively immunoreacted with the ubiquitinated inclusions (not shown). Similar to ubiquitinated epitopes, the presence of SEDI immunoreactive inclusions in proximal dendrites or cell body in all occasions was associated with Golgi fragmentation (Figure 2D).

To further establish the relationship between Golgi fragmentation and the presence of ubiquitin-positive inclusions, we analyzed Golgi fragmentation in spinal cord section of T3hSOD1 mice that express a relatively low level of G93A mutant SOD1 specifically in neurons and, in addition, ubiquitously express high levels of wild-type SOD1 [31]. T3hSOD1 mice develop signs of muscle weakness starting from 1 year of age, while dendritic ubiquitinated inclusions are already present in a small number of motor neurons at the age of 20 weeks [31,38]. T3hSOD1 showed fragmented Golgi apparatus in a subset of motor neurons. The number of neurons with fragmented Golgi increased with aging, and a subset of these also showed ubiquitin positive inclusions in the cell body (Figure 2E-G). Importantly, similar to G1del mice, all motor neurons in T3hSOD1 mice with ubiquitin-positive inclusions in the cell body or proximal dendrites showed fragmented Golgi apparatus, while inversely, only a subset of T3hSOD1 motor neurons with fragmented Golgi apparatus show ubiquitin-positive inclusions in the cell body or proximal dendrites.

### Golgi fragmentation does not depend on ATF3 expression

**Figure 3:**
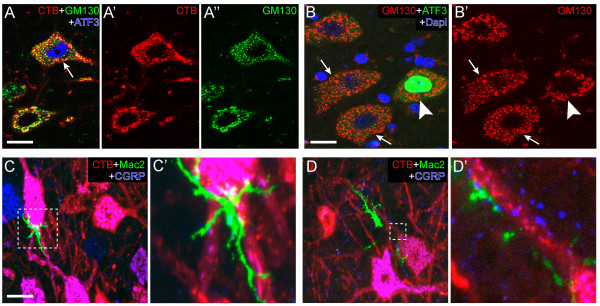
**CTB tracing and Golgi fragmentation in motor neurons expressing ATF3 or contacted by phagocytosing microglia. A)** Confocal image showing a CTB-positive motor neuron with fragmented Golgi and ATF3-positive nucleus (arrow). **B)** Confocal image of motor neurons with fragmented Golgi that are ATF3-negative (arrows) and a motor neuron with a normal Golgi apparatus that is ATF3-positive. **C**, **D)** Maximal projections of confocal stacks showing CTB-positive motor neurons contacted by Mac-2 positive microglial cells. Bars: 20 μm **(A-C)**.

Double-labeling with CTB with an antibody against ATF3 or activated phagocytosing microglia (Mac-2 positive, [38]) revealed CTB-positive motor neurons that expressed ATF3 or were contacted by phagocytosing microglia (Figure 3), indicating that also these pathological signatures precede distal axon degeneration. Double-labeling of ATF3 and GM130 showed that consistent with previous data [29] most (>80%) ATF3-positive motor neurons also have fragmented Golgi (Figure 3A). Inversely, however, many motor neurons with fragmented Golgi were ATF3-negative (Figure 3B). Counting of lumbar sections of G1del mice aged 20 (n = 3) and 25 weeks (n = 3) indicated ATF3 expression in 40 ± 6 (25–62) % (Mean ± SE, range) and 54 ± 13 (37–75) % of motor neurons with Golgi fragmentation. Analysis of ATF3 and ubiquitin double labeled sections indicated that all motor neurons with ubiquitinated aggregates in the cell body are ATF3 positive. Since motor neurons with ubiquitinated aggregates always show Golgi fragmentation, together the data suggest that Golgi fragmentation occurs independently from ATF3 expression in a significant proportion of motor neurons, but codistributes with ATF3 in motor neurons with ubiquitinated aggregates.

### Golgi fragmentation in SOD-ALS mice does not depend on apoptotic pathways

**Figure 4:**
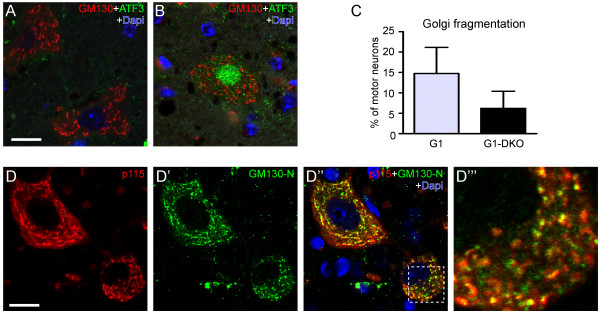
**Golgi fragmentation in SOD1-ALS mice is not downstream of apoptotic pathways. A**, **B)** Confocal images of GM130 and ATF3 labeling in spinal motor neurons of a 90 day old fast SOD1-G93A mouse (G1 line) crossed into mouse deficient for BAK in all cells and BAX specifically in neurons (DKO) resulting in deficiency of the mitochondrial apoptotic pathways. Note, motor neurons with normal (A) and fragmented **(B)** Golgi apparatus and ATF3 expression in motor neurons with fragmented Golgi. **C)** G1-DKO showed a lower proportion Golgi fragmented motor neurons but this difference was not significant (unpaired Student’s *t*-test); values are means ± SE (n = 3 mice per group). **D)** Confocal image of p115 and GM130 (N-terminus) double labeling in motor neurons in 25 week old G1del mouse showing that motor neurons with fragmented Golgi (insert) show the same relative intensity of p115 staining. Bar: 10 μm.

There is substantial evidence of early activation of apoptotic pathways in SOD1-ALS mice [49,50], and accordingly complete blockade of the mitochondrial apoptotic pathway through deletion of both BAX and BAK attenuated motor neuron loss and extended survival in high copy (fast) G1 mice [33]. Analysis of archival paraffin spinal cord sections from 90 days old G1 BAX-BAK double KO mice from the study by Reyes et al. [33] revealed that these mice still show Golgi fragmentation (Figure 4A-C), indicating that Golgi fragmentation in G1 mice does not depend on a functional mitochondrial apoptotic pathway.

Golgi fragmentation in apoptosis may depend on caspase 3 mediated cleavage of several membrane tethering factors involved in the structural organization of the Golgi, in particular GRASP65 and p115 [16,51,52]. We therefore examined whether motor neurons with Golgi fragmentation show reduced expression of these proteins. However, there was no evidence for a relative reduction of GRASP65 (not shown) or p115 (Figure 4D) expression in Golgi fragmented motor neurons. Together the data suggest no major role of apoptotic pathways in triggering Golgi fragmentation in G1 mice motor neurons.

### Golgi fragmentation is facilitated in SOD1-ALS mice with reduced dynein/dynactin function

**Figure 5:**
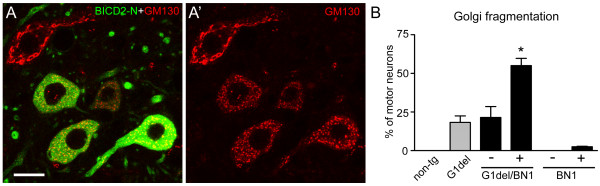
**Facilitated Golgi fragmentation in mice G1del motor neurons with impaired dynein function. A)** Representative confocal image of L4 lumbar motor neurons of a BN1/G1del transgenic mouse of 28 weeks of age. BN1 mice express GFP-BICD2-N in 50-60% of the motor neurons [17]. Note, Golgi fragmentation in GFP-BICD2-N neurons and normal Golgi apparatus in the other motor neurons. Bar, 20 μm. **B)** Bar graph showing the proportion of motor neurons with Golgi fragmentation in cervical C6/7 motor neurons of G1del, BN1, and G1del/BN1 mice of 28 weeks of age. Note, a much larger proportion of motor neurons with Golgi fragmentation in GFP-BICD2-N-positive (+) motor neurons of G1del/BN1 mice as compared to GFP-BICD2-N-negative (-) motor neurons of G1del/BN1 mice and motor neurons of G1del mice, which show similar percentages of motor neurons with Golgi fragmentation. *, P < 0.01, compared to G1del and G1del/BN1- motor neurons, Tukey’s multiple comparison test after one-way ANOVA. The difference in relative proportion of Golgi fragmentation can not be explained by differential degeneration of GFP-BICD2-N-positive versus GFP-BICD2-N-negative neurons, as at this age there is minimal loss of cervical motor neurons in G1del mice (Additional file 1: Figure S2).

Fragmentation and dispersion of the Golgi apparatus is a well established consequence of inhibition of the dynein/dynactin microtubule motor complex [53-55]. Accordingly, we showed that impairment of dynein function via overexpression of the N-terminus of Bicaudal D2 (BICD2-N) causes Golgi fragmentation in cultured neurons and in *vivo* in motor neurons of transgenic BICD2-N mice [17,56]. Here we have analyzed the occurrence of Golgi fragmentation in double transgenic G1del/BICD2-N mice resulting from crossing of G1del mice with BICD2-N mice from the BN1 line. Of note, BN1 mice show expression of the BICD2-N transgene in 50-60% of the motor neurons, and show Golgi fragmentation only in a small proportion (1-10% depending on the age) of transgenic motor neurons [17]. Significantly, more than 50% of BICD2-N-expressing motor neurons showed Golgi fragmentation in G1del/BN1 mice (Figure 5), which is a much larger proportion than in BICD2-N-negative motor neurons in the same mice, and in motor neurons of G1del littermates (Figure 5B). These data indicate that impaired dynein/dynactin function facilitates the process causing Golgi fragmentation in G1del mice, or vice versa, and suggest that Golgi fragmentation in G1del mice is related to impaired dynein-dynactin dependent trafficking.

### Reduced numbers of endosomes in cells with Golgi fragmentation

**Figure 6:**
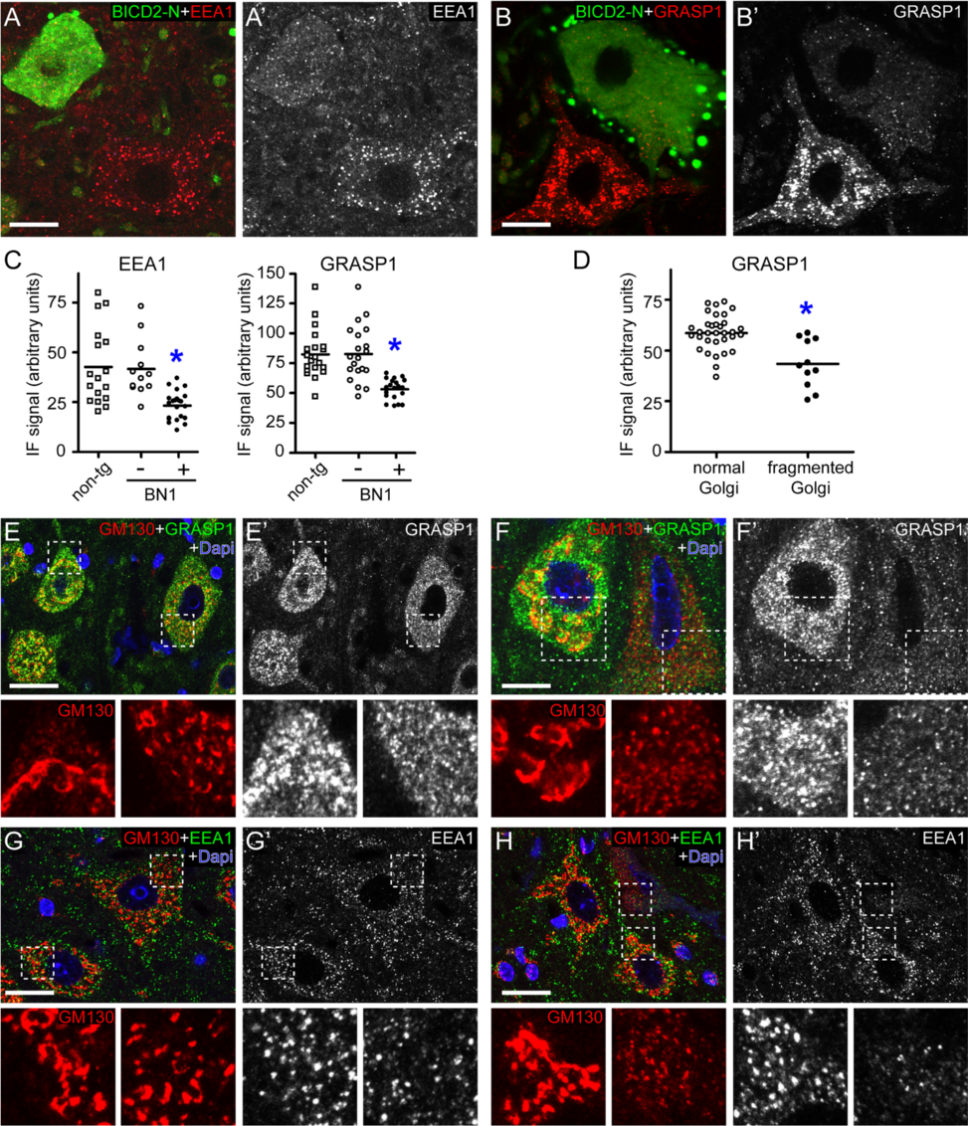
**Reduced endosome levels in motor neurons with Golgi fragmentation. A**, **B)** Confocal images of staining of the endosomal proteins EEA1 **(A)** and GRASP1 **(B)** in lumbar spinal cord sections of a BN1 transgenic mouse. Note reduced EEA1 and GRASP1 labeling in GFP-BICD2-N positive neurons. **C)** Analysis of fluorescent intensities of GFP-BICD2-N positive versus negative motor neurons showed reduced mean intensities in GFP-BICD2-N positive motor neurons (P < 0.05, unpaired Student's t-test negative (-) versus positive (+) neurons). Note that all values were collected from non-transgenic (n = 2 mice) and BN1 (n = 4) lumbar spinal cord specimen embedded in a single gelatin block and processed in the same immunorun (see Material and methods). **D-F)** Quantitation **(D)** and representative images **(E**, **F)** of GRASP1-labeling in motor neurons with normal and fragmented Golgi showing reduced GRASP1 labeling in motor neurons with fragmented Golgi (P < 0.05, unpaired Student's t-test, normal versus fragmented Golgi). **G**, **H)** Confocal images of EEA1-labeling in motor neurons with normal and fragmented Golgi showing reduced EEA1 labeling in motor neurons with fragmented Golgi. Bars, 10 μm.

Inhibition of dynein-dynactin function, in addition to Golgi fragmentation also causes relocalization of other organelles, in particular endosomes that in conditions of impaired dynein-dynactin function redistribute to the cell periphery in non-polarized cells [53-55]. We therefore studied the distribution of endosomes in motor neurons of BN1 and G1del mice using antibodies against early endosome antigen 1 (EEA1) [57] and Rab4 binding protein GRASP-1 [58,59]. Strikingly, instead of a redistribution of endosomes to the cell periphery, BICD2-N-expressing motor neurons in BN1 mice showed an overall reduction of endosomal labeling in both EAA1 and GRASP1 stained sections (Figure 6A-C). The same was observed in motor neurons with Golgi fragmentation in G1del mice which showed reduced levels of GRASP1 and EEA1 staining that correlated with the severity of Golgi fragmentation (Figure 6D-H). In both BICD2-N and G1del mice, changes in endosomal labelling were most evident in sections stained for GRASP-1, as reduced labelling was associated with a loss of the relatively large particles. These data indicate that motor neurons with Golgi fragmentation in G1del mice reproduce an additional organelle abnormality that also occurs in motor neurons with impaired dynein/dynactin function.

### Reduced levels of endosomes in neurons after inhibition of dynein/dynactin

**Figure 7:**
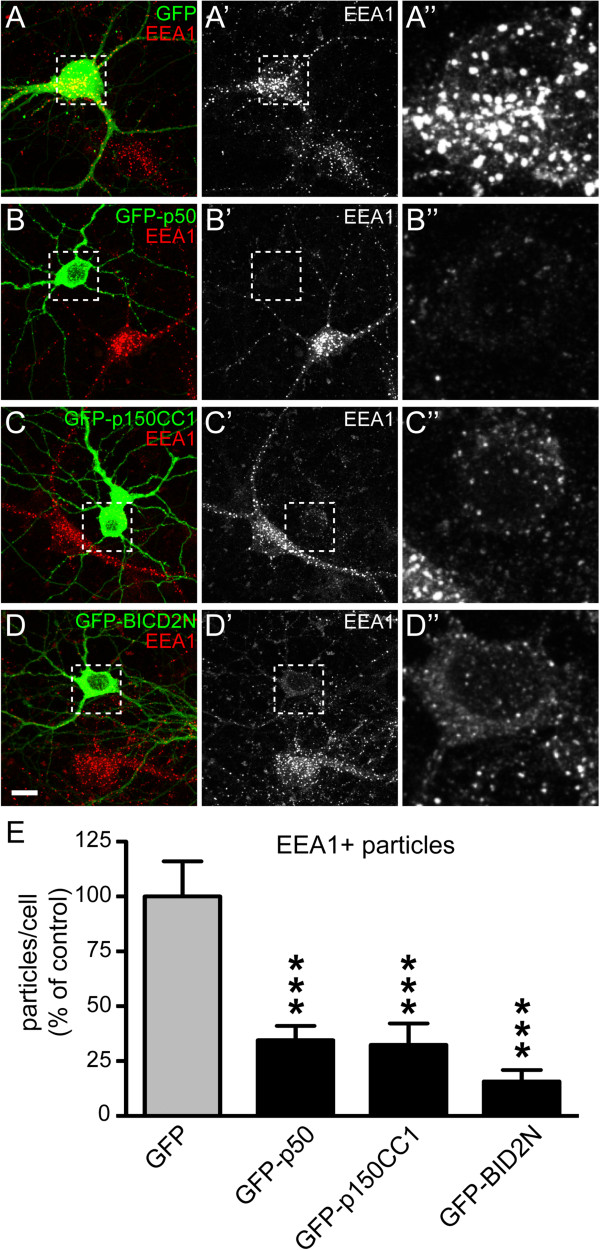
**Reduced endosome levels after dynein-dynactin inhibition. A-D)** Primary hippocampal neurons expressing the dynactin subunit p50 (GFP-p50), a dominant-negative dynactin construct (GFP-p150-cc1) or GFP-BICD2-N, show reduced EEA1 labelling and a lower EEA1 particle density compared to GFP transfected and untransfected neurons **(A)**. Inset shows enlargement of soma. Bar: 10 μm. **E)** Bar graph of the relative particle density. ***, P < 0.001 compared to GFP group, Tukey’s multiple comparison test following one-way ANOVA (P < 0.001).

The above data indicate a different effect of dynein/dynactin inhibition on endosome distribution in neurons versus non-polarized cells. To further substantiate these differences, we examined the effect of dynein/dynactin inhibition on the distribution of endosomes in primary cultured hippocampal neurons. In accord with reduced endosomal labelling in motor neurons of BICD2-N mice, inhibition of dynein-dynactin function in primary cultured hippocampal neurons by overexpression of BICD2-N, the dynactin subunit p50, or a dominant-negative dynactin construct (p150-cc1) [17,39,53] also resulted in reduced labelling of the endosomal marker EEA1, rather than a redistribution of endosomes to the cell periphery (Figure 7). These data support the notion that reduced endosomal labelling in motor neurons with Golgi fragmentation in G1del mice may reflect impaired dynein/dynactin function.

## Discussion

Golgi fragmentation is a well-established feature of ALS spinal and cortical motor neurons, that also occurs in some other neurodegenerative conditions, is reproduced in SOD1-ALS mice and some cellular ALS models [6,60], but is not found in degenerating motor neuron in a non-ALS disorder [61] and non-ALS mouse models [62]. Golgi fragmentation in ALS and ALS mouse models also differs from the large variety of Golgi apparatus morphological abnormalities that we observed in motor neurons of a DNA-repair deficient mouse model [63]. In motor neurons of ALS patients, the presence of Golgi fragmentation correlates with an abnormal TDP-43 distribution or the presence of inclusions, suggesting a relationship between Golgi fragmentation and the presence of protein aggregates [22-24]. Accordingly, here we show in SOD1-ALS mice that Golgi fragmentation occurs in all motor neurons with ubiquitinated SOD1 inclusions. However, a major portion of motor neurons with Golgi fragmentation did not show such inclusions. This can be explained by the presence of inclusions in dendrites that were not in the plane of the section, or small aggregates that are not detectable at the light microscopic level [64]. Alternatively, Golgi fragmentation may be caused by a process upstream of large aggregates, perhaps mediated by toxic misfolded or oligomeric SOD1 species [64,65]. Using antibodies against misfolded and aggregated SOD1 species (USOD and SEDI, [36,37]), we did not detect changes that showed a consistent correlation with Golgi fragmentation.

In the present study, we also show that motor neurons with Golgi fragmentation more frequently display Golgi apparatus within the proximal dendrite. Despite the limitations of our analysis, in that we only analyzed the dendrites in the transverse plane, the data indicate that the likelihood for a proximal dendrite showing Golgi apparatus is 3 times higher in cells with Golgi fragmentation. Data from hippocampal neurons indicate that the Golgi apparatus orients toward the longest dendrite, and that the presence of the Golgi apparatus and Golgi outposts (i.e. discrete compartments that are discontinuous with somatic Golgi) into dendrites relates to local secretory demands [9,66,67]. Thus the increased presence of Golgi apparatus in dendrites and perhaps also Golgi fragmentation may reflect a reorganization of the secretory pathway in response to increased secretory demands from specific dendrites, e.g. to repair dendritic damage.

Several findings suggest that Golgi fragmentation in SOD1-ALS motor neurons is associated with reorganized or aberrant trafficking: First, fragmentation into ministacks is reminiscent of Golgi fragmentation resulting from impaired dynein-dependent transport [6,17]. Second, a subset of neurons with Golgi fragmentation, in spite of their ability of take up, retrogradely transport, and redistribute the CTB throughout the somatodendritic compartment, do not show accumulation of CTB in the trans-Golgi, suggestive of impaired endosome to Golgi retrograde trafficking. Third, we show a large increase in Golgi fragmentation in G1del mouse crossed with a mouse model showing impaired dynein/dynactin dependent transport, indicative of a synergistic interaction between the process causing Golgi fragmentation and dynein-dynactin dependent trafficking. Fourth, we show that motor neurons with Golgi fragmentation like motor neurons with impaired dynein/dynactin function show reduced expression of endosomal markers. Of note, the endosomal changes that we observed in motor neurons with impaired dynein/dynactin function are different from those observed in cultured non-neuronal cells with radially oriented microtubules. The differences between neurons and other cells may follow from differences in microtubule organization as well as differences in endosomal functional pathways [68,69].

Previous data from BICD2-N transgenic mice suggested Golgi fragmentation is not necessarily detrimental [17]. Furthermore, we showed that G1del mice crossed with BICD2-N transgenic mice show increased life span, which combined with the finding of the present study that these mice show increased levels of Golgi fragmentation, suggests that Golgi fragmentation is not detrimental and perhaps reversible in SOD1-ALS mice. A recent study demonstrates reversible Golgi fragmentation in hippocampal neurons cultured in hyperexcitable conditions [18]. Reversible Golgi fragmentation has also been shown to be part of a cytoprotective DNA damage response, where DNA damage protein kinase, DNA-PK, via phosphorylation of the Golgi protein GOLPH3 enhances the interaction between GOLPH3 and the myosin MYO18A and facilitates Golgi vesiculation [19]. The extent to which Golgi fragmentation in ALS motor neurons also is part of a coordinated stress response, that for instance is triggered by the presence of protein aggregates, remains to be established.

Multiple pathological events have been identified in SOD1-ALS mice models, including mitochondrial abnormalities, ER stress, proteotoxic stress, abnormal ion and glutamate homeostasis, abnormal axonal transport, axonal degeneration, and non-cell autonomous toxic events mediated by glial cells, but the relationship between these events, and the extent to which they occur in SOD1-ALS and other ALS patients remains to be established [70]. Golgi fragmentation is a well established feature of ALS motor neurons. In the present study we confirm that Golgi fragmentation is a very early event that precedes ATF3 induction and degeneration of the distal axon, and occurs independently of the activation of apoptosis pathways. These data, for instance, challenge the notion that ALS primarily is a dying back axonopathy as suggested by some studies [40-42].

## Conclusions

Together the data indicate that Golgi fragmentation is a very early event in the pathological cascade in ALS that is associated with altered organization of intracellular trafficking. We propose that Golgi fragmentation is an early response to deleterious mechanisms shared by several ALS forms. Further identification of the mechanisms upstream of Golgi fragmentation will provide important insights in ALS pathogenesis.

## Competing interests

The authors declare that they have no competing interests.

## Authors’ contributions

VvD, MK, CCH and DJ designed research; VvD, MK, EDH and ET performed experiments, VvD, MK, EDH, ET and DJ analyzed data; SAO provided histological material; VvD, MK, and DJ wrote the paper; CCH and DJ supervised the project. All authors read and approved the final manuscript.

## Supplementary Material

Additional file 1: Table S1 Number and % of proximal dendrites with Golgi apparatus in motor neurons with or without Golgi fragmentation in G1del SOD1-G93A mice. **Figure S1**. A larger proportion of dendrites with Golgi apparatus in motor neurons with Golgi fragmentation. Retrogradely CTB-GM130 double labeled motor neurons were sampled in lumbar L4 transverse sections from three G1del mice of 20 weeks, on the basis of the presence of at least 3 dendrites within the plane of section with at least 2 dendrites that could be followed to the first branching point. Confocal stacks of CTB and GM130 stacks were collected, and cells were analyzed for the occurrence of Golgi fragmentation and the presence of dendritic GM130 labeling. Bar graphs are based on 24 and 14 motor neurons with normal and fragmented Golgi apparatus, respectively. The mean number of dendrites in the plain of sections was the same for motor neurons with normal versus fragmented Golgi apparatus. However, the proportion of dendrites with Golgi apparatus was higher in cells with fragmented Golgi *, P < 0.01, Student’s *t*-test. **Figure S2**. Bar graph showing the number of ChAT-labelled motor neurons in cervical C6/7 motor neurons of G1del, BN1, G1del/BN1 and non-transgenic mice aged 28 weeks. (see [17] for Materials and methods). Click here for file
