# Supplementary material for: Golgi fragmentation precedes neuromuscular denervation and is associated with endosome abnormalities in SOD1-ALS mouse motor neurons
Source: Acta Neuropathol Commun. 2014 Apr 7;2:38. doi: 10.1186/2051-5960-2-38 (PMC4023628; doi:10.1186/2051-5960-2-38)
Supplement: Additional file 1: Table S1 — Number and % of proximal dendrites with Golgi apparatus in motor neurons with or without Golgi fragmentation in G1del SOD1-G93A mice. Figure S1. A larger proportion of dendrites with Golgi apparatus in motor neurons with Golgi fragmentation. Retrogradely CTB-GM130 double labeled motor neurons were sampled in lumbar L4 transverse sections from three G1del mice of 20 weeks, on the basis of the presence of at least 3 dendrites within the plane of section with at least 2 dendrites that could be followed to the first branching point. Confocal stacks of CTB and GM130 stacks were collected, and cells were analyzed for the occurrence of Golgi fragmentation and the presence of dendritic GM130 labeling. Bar graphs are based on 24 and 14 motor neurons with normal and fragmented Golgi apparatus, respectively. The mean number of dendrites in the plain of sections was the same for motor neurons with normal versus fragmented Golgi apparatus. However, the proportion of dendrites with Golgi apparatus was higher in cells with fragmented Golgi *, P < 0.01, Student’s t-test. Figure S2. Bar graph showing the number of ChAT-labelled motor neurons in cervical C6/7 motor neurons of G1del, BN1, G1del/BN1 and non-transgenic mice aged 28 weeks. (see [17] for Materials and methods). [file 2051-5960-2-38-S1.pdf]

Supplementary Data of ' Golgi fragmentation precedes neuromuscular denervation and is associated with endosome abnormalities in SOD1-ALS mouse motor neurons '

Vera van Dis, Marijn Kuijpers, Elize D. Haasdijk, Eva Teuling, Scott A. Oakes, Casper C. Hoogenraad, Dick Jaarsma

Number of Supplementary Tables: 1

Number of Supplementary Figures: 2

Table S1:Number and % of proximal dendrites with Golgi apparatus in motor neurons with or without Golgi fragmentation in G1del SOD1-G93A mice

normal GA					fragmented GA			
	# of dendrites*					# of dendrites*		
	**total**	**with GA**	**%**			**total**	**with GA**	**%**
cell 1	3	2	67		cell 1	6	5	83
cell 2	5	0	0		cell 2	3	1	33
cell 3	3	0	0		cell 3	4	2	50
cell 4	3	0	0		cell 4	4	2	50
cell 5	3	0	0		cell 5	3	0	0
cell 6	4	0	0		cell 6	3	2	67
cell 7	4	2	50		cell 7	3	1	33
cell 8	3	2	67		cell 8	4	1	25
cell 9	4	0	0		cell 9	3	2	67
cell 10	3	0	0		cell 10	4	2	50
cell 11	3	0	0		cell 11	3	2	67
cell 12	3	1	33		cell 12	4	0	0
cell 13	4	1	25		cell 13	5	2	40
cell 14	4	1	25		cell 14	3	1	33
cell 15	3	0	0					
cell 16	3	0	0					
cell 17	3	0	0					
cell 18	5	1	20					
cell 19	3	1	33					
cell 20	3	0	0					
cell 21	4	0	0					
cell 22	4	1	25					
cell 23	3	0	0					
cell 24	3	1	33					
**Mean**	**3,46**	**0,541**	**16**			**3,71**	**1,64**	**43**
Total	83	13				52	23	

*, As the analyses was performed in transverse 40 µm thick sections only the subset of dendrites within the section could be analyzed. See material and methods for further details.

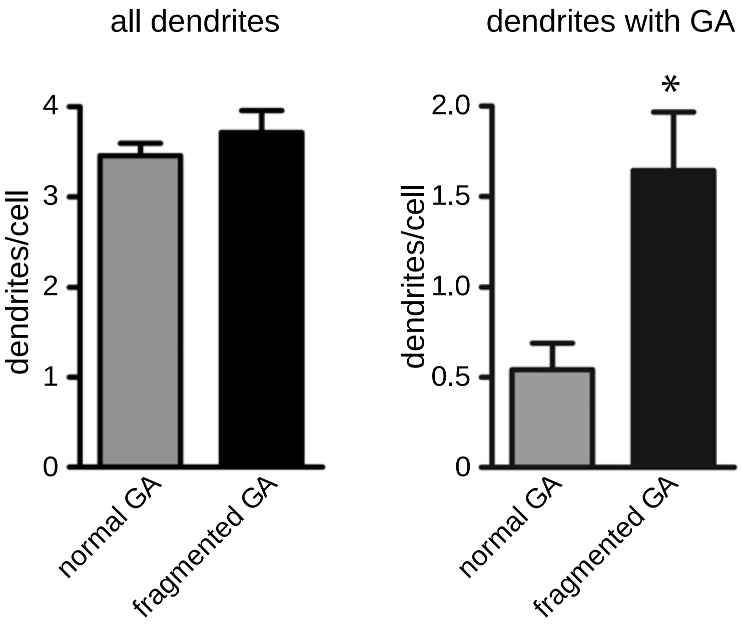

Figure S1. A larger proportion of dendrites with Golgi apparatus in motor neurons with Golgi fragmentation

Retrogradely CTB-GM130 double labeled motor neurons were sampled in lumbar L4 transverse sections from three G1del mice of 20 weeks, on the basis of the presence of at least 3 dendrites within the plane of section with at least 2 dendrites that could be followed to the first branching point. Confocal stacks of CTB and GM130 stacks were collected, and cells were analyzed for the occurrence of Golgi fragmentation and the presence of dendritic GM130 labeling. Bar graphs are based on 24 and 14 motor neurons with normal and fragmented Golgi apparatus, respectively. The mean number of dendrites in the plain of sections was the same for motor neurons with normal versus fragmented Golgi apparatus. However, the proportion of dendrites with Golgi apparatus was higher in cells with fragmented Golgi *, $P < 0.01$, Student's *t*-test.

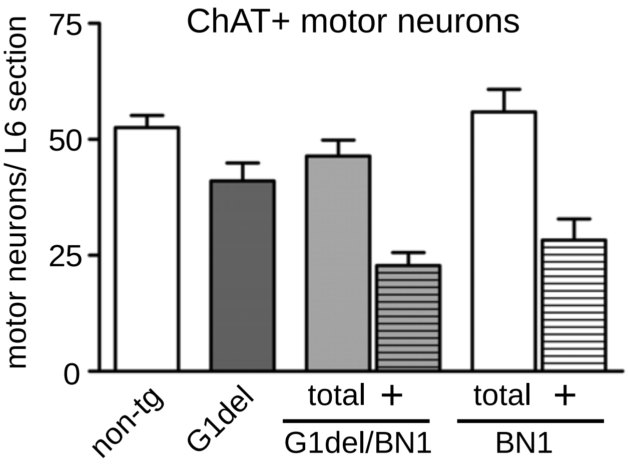

Figure S2. Bar graph showing the number of ChAT-labelled motor neurons in cervical C6/7 motor neurons of G1del, BN1, G1del/BN1 and non-transgenic mice aged 28 weeks. (see [17] for materials and methods).
